# IL-22-dependent responses and their role during *Citrobacter rodentium* infection

**DOI:** 10.1128/iai.00099-24

**Published:** 2024-04-01

**Authors:** Karine Melchior, Romana R. Gerner, Suzana Hossain, Sean-Paul Nuccio, Cristiano Gallina Moreira, Manuela Raffatellu

**Affiliations:** 1 Division of Host-Microbe Systems and Therapeutics, Department of Pediatrics, University of California San Diego, La Jolla, California, USA; 2 School of Pharmaceutical Sciences, São Paulo State University (UNESP), Araraquara, São Paulo, Brazil; 3 School of Life Sciences, ZIEL – Institute for Food and Health, Freising-Weihenstephan, Technical University of Munich, Munich, Germany; 4 Department of Internal Medicine III, University Hospital rechts der Isar, Technical University of Munich, Munich, Germany; 5 Department of Biological Sciences, Louisiana State University, Baton Rouge, Louisiana, USA; 6 Center for Microbiome Innovation, University of California San Diego, La Jolla, California, USA; 7 Chiba University-UC San Diego Center for Mucosal Immunology, Allergy, and Vaccines (CU-UCSD cMAV), La Jolla, California, USA; University of California, Davis, Davis, California, USA

**Keywords:** *Citrobacter*, mucosal immunity, gut inflammation, antimicrobial peptides

## Abstract

The mouse pathogen *Citrobacter rodentium* is utilized as a model organism for studying infections caused by the human pathogens enteropathogenic *Escherichia coli* (EPEC) and enterohemorrhagic *E. coli* (EHEC) and to elucidate mechanisms of mucosal immunity. In response to *C. rodentium* infection, innate lymphoid cells and T cells secrete interleukin (IL)-22, a cytokine that promotes mucosal barrier function. IL-22 plays a pivotal role in enabling mice to survive and recover from *C. rodentium* infection, although the exact mechanisms involved remain incompletely understood. Here, we investigated whether particular components of the host response downstream of IL-22 contribute to the cytokine’s protective effects during *C. rodentium* infection. In line with previous research, mice lacking the IL-22 gene (*Il22*^−/−^ mice) were highly susceptible to *C. rodentium* infection. To elucidate the role of specific antimicrobial proteins modulated by IL-22, we infected the following knockout mice: *S100A9*^−/−^ (calprotectin), *Lcn2*^−/−^ (lipocalin-2), *Reg3b*^−/−^ (Reg3β), *Reg3g*^−/−^ (Reg3γ), and *C3*^−/−^ (C3). All knockout mice tested displayed a considerable level of resistance to *C. rodentium* infection, and none phenocopied the lethality observed in *Il22*^−/−^ mice. By investigating another arm of the IL-22 response, we observed that *C. rodentium*-infected *Il22^−/^*^−^ mice exhibited an overall decrease in gene expression related to intestinal barrier integrity as well as significantly elevated colonic inflammation, gut permeability, and pathogen levels in the spleen. Taken together, these results indicate that host resistance to lethal *C. rodentium* infection may depend on multiple antimicrobial responses acting in concert, or that other IL-22-regulated processes, such as tissue repair and maintenance of epithelial integrity, play crucial roles in host defense to attaching and effacing pathogens.

## INTRODUCTION

Gastrointestinal infections caused by bacterial pathogens are associated with substantial morbidity and mortality worldwide (1). Among diarrheal pathogens, the attaching and effacing (A/E) bacteria enteropathogenic *Escherichia coli* (EPEC) and enterohemorrhagic *E. coli* (EHEC) stand out as major public health concerns (2, 3). Because mice exhibit relatively high resistance to EHEC and EPEC infection, the A/E mouse pathogen *Citrobacter rodentium* has become a widely used model for investigating the mechanisms, by which A/E pathogens cause disease, and for gaining insights into mucosal responses during infection (4, 5).

Interleukin (IL)-22 is a cytokine that plays a central role in orchestrating various aspects of gut mucosal immunity. It is produced by lymphocyte subsets in response to proinflammatory signals, including IL-23, which is produced by myeloid cells (6). Lymphocytes that secrete IL-22 include Th1, Th22, CD8^+^ T cells, γδ T cells, natural killer cells, lymphoid tissue inducer cells, and innate lymphoid type 3 cells (ILC3) (7, 8). During infection, IL-22 confers protection to the host by stimulating epithelial cells to produce antimicrobial proteins, including the C-type lectins Reg3β and Reg3γ, which exhibit direct antimicrobial activity against a subset of bacterial pathogens (7, 9–13). IL-22 also orchestrates nutritional immunity, a host defense strategy that restricts pathogen access to essential metal nutrients (14, 15), by inducing expression of the metal-sequestering antimicrobial proteins calprotectin, lipocalin-2, and hemopexin (9, 16–20). Additionally, IL-22 has been shown to induce the expression of complement component C3 (19) and to regulate critical aspects of intestinal epithelial barrier function, including gut permeability and regeneration (7).

The protective role of IL-22 during infection varies depending on the context and the pathogen involved. For example, the absence of IL-22 does not increase host susceptibility during infection with *Neisseria gonorrhoeae*, *Listeria monocytogenes*, or *Salmonella enterica* serovar Typhimurium (21–24). In contrast, IL-22 is essential for protecting the host against *Klebsiella pneumoniae* and *C. rodentium* (9, 25). When infected with *C. rodentium*, wild-type (WT) C57BL/6 mice do not develop overt disease and clear the infection within a couple of weeks. However, C57BL/6 *Il22*^−/−^ mice succumb to the infection, similar to *C. rodentium*-susceptible mouse lines such as C3H/HeJ mice (26, 27).

Several IL-22-dependent antimicrobial defenses have been associated with protection during *C. rodentium* infection, including the regulation of antimicrobial proteins (9, 16) and gut epithelial function (28, 29). In this study, we aimed to investigate the specific contribution of some IL-22-dependent antimicrobial responses to host protection during *C. rodentium* infection. Despite C57BL/6 *Il22*^−/−^ mice showing high susceptibility to *C. rodentium* infection, all C57BL/6 knockout mice that we tested, including *S100A9*^−/−^ mice (lacking calprotectin), *Lcn2*^−/−^ mice, (lacking lipocalin-2), *Reg3b*^−/−^ mice (lacking Reg3β), *Reg3g*^−/−^ mice (lacking Reg3γ), and *C3*^−/−^ mice (lacking C3), demonstrated resistance to lethal *C. rodentium* infection comparable to WT mice. Our results indicate that resistance to *C. rodentium* infection may depend on multiple antimicrobial responses acting in concert or that other IL-22-regulated processes, such as tissue repair and maintenance of epithelial integrity, play important roles in IL-22-mediated host defense to A/E pathogens.

## MATERIALS AND METHODS

### Bacterial strains

*C. rodentium* strain DBS100 Nal^R^ (a fully virulent, spontaneous nalidixic acid (Nal)-resistant derivative of WT strain DBS100; obtained from Prof. Vanessa Sperandio, University of Wisconsin, Madison) was used in this study (30). Bacteria were routinely cultured at 37°C overnight aerobically in Luria-Bertani (LB; Miller) broth with rotation or on LB agar (1.5% wt/vol) plates containing Nal (50 µg/mL). Overnight cultures were centrifuged, washed, and resuspended in 1× phosphate-buffered saline (PBS; pH 7.4) to a concentration of 5 × 10^9^ colony-forming units (CFUs) per 200 µL for inoculation.

### Animal experiments

All infection experiments utilized 5-week-old male and female C57BL/6 mice. *Il22^−/^*^−^ (6), *Lcn2^−/^*^−^ (20), *S100a9^−/^*^−^ (31), *C3^−/^*^−^ (the Jackson Laboratory, stock #029661) (32), *Reg3b^−/^*^−^ (33), and *Reg3g^−/^*^−^ (13) mice (all in the C57BL/6 background) were orally gavaged with 5 × 10^9^ CFU *C. rodentium* in 200-µL 1× PBS. *Il22^−/^*^−^ mice were obtained from *Il22*^+/−^ heterozygous breeders and were cohoused with their WT littermates from weaning and through the course of infection. For all other colonies, knockout mice were obtained from knockout breeders and were cohoused with WT mice (purchased from the Jackson Laboratory) from a week before infection (i.e., at 4 weeks of age) and through the course of infection. All mice were free of detectable *E. coli* in their fecal samples, based on MacConkey agar plating. Fecal samples were collected on Days 1, 4, 7, 10, and 12 of post-infection, homogenized, serially diluted, and plated on LB + Nal agar for CFU enumeration. Mice were monitored for 25 days of post-infection. A loss of 20% from initial body weight or moribund behavior/appearance was used as a humane endpoint. Some mice were euthanized at Day 12 of post-infection for histopathology and gene expression analysis. All animal experiments were reviewed and approved by the Institutional Animal Care and Use Committee at the University of California, San Diego.

### RNA extraction

Colons collected from mice infected with *C. rodentium* and from uninfected controls were snap-frozen in liquid nitrogen and then stored at −80°C until further processing. The samples were homogenized with a mortar and pestle, collected in an RNAse-free 1.5-mL tube containing TRI Reagent (Millipore-Sigma), vortexed for 5 minutes, transferred to a microfuge tube containing 0.1 mL of 1-bromo-3-chloropropane (Molecular Research Center), vortexed, incubated at room temperature for 10 minutes, and then centrifuged at 12,000 × *g* for 15 minutes at 4°C. To precipitate RNA, the aqueous phase was then transferred to a tube with 0.5 mL of isopropanol (Fisher Chemical), incubated for 30 minutes at −20°C, and then centrifuged at 12,000 × *g* for 15 minutes at 4°C. The RNA was then washed with 75% ethanol (Fisher Chemical) and centrifuged at 12,000 × *g* for 8 minutes at 4°C. Ethanol was removed by decanting, and the tubes were allowed to air-dry, after which the RNA pellets were dissolved in ultrapure water (Invitrogen; DNase- and RNase-free) and the RNA concentration was measured.

### Analysis of gene expression by qRT-PCR

Reverse transcription of 1 µg of total RNA was performed using the SuperScript IV VILO with ezDNase cDNA Synthesis Kit (Thermo Fisher Scientific). Quantitative real-time PCR (qRT-PCR) for gene expression analysis was performed using the PowerUp SYBR Green Master Mix (Thermo Fisher Scientific) on a QuantStudio 5 Real-Time PCR System (Thermo Fisher Scientific). Host gene expression was normalized to β-actin (*Actb*). Fold changes in gene expression were relative to the respective uninfected controls (WT or *Il22*^−/−^) and calculated using the ΔΔCt method. The primers used in the study are listed in Table S1.

### FITC-Dextran gut permeability assay

Five-week-old male and female *Il22*^+/+^ and *Il22*^−/−^ littermate mice were infected with *C. rodentium* as described. At Day 12 of post-infection, mice were orally gavaged with 100 µL of 80-mg/mL fluorescein isothiocyanate-dextran (FITC-dextran; average molecular weight of 4,000; Millipore-Sigma) in 1× PBS. After 4 hours, mice were euthanized, and blood was collected by cardiac puncture and transferred to microfuge tubes containing 0.5-M EDTA. Spleen and fecal samples were collected for CFU enumeration. Tubes with blood were centrifuged, and then plasma was collected and diluted in a ratio of 1:4 in 1× PBS. PBS was used as a blank to normalize the data. Fluorescence was determined at 530 nm with an excitation of 485 nm on a BioTek Synergy HTX plate reader.

### Histopathology

At Day 12 of post-infection, whole colons from infected WT and *Il22*^−/−^ mice were processed into Swiss rolls, formalin-fixed, paraffin-embedded, and sectioned for blinded evaluation of histopathology. The following parameters were scored as follows: mononuclear infiltration scores are 0 for absent (normal sparse lymphocytic infiltrate), 1 for mild (diffuse increase in lamina propria, usually with plasma cells), 2 for moderate (lamina propria increased with basal localization aggregates displacing crypts distinguish normal mucosa-associated lymphoid tissue), and 3 for severe (lamina propria with submucosal infiltration); crypt hyperplasia scores are 0 for absence and 3 for the highest score (severe); epithelial injury scores are 1 for mild (crypt dropout/crypt distortion or surface epithelial damage, no frank erosion or ulceration), 2 for moderate (focal ulceration), and 3 for severe (multifocal or extensive ulceration); neutrophilic crypt abscesses scores are 0 for absent, 1 for mild (lamina propria only), 2 for moderate (lamina propria plus cryptitis and crypt abscesses), and 3 for severe (sheet-like or submucosal infiltrate).

### Statistical analysis

Statistical analysis was performed with GraphPad Prism v9. The Shapiro–Wilk test was used for normality distribution. For normally distributed groups, *P* values were calculated by unpaired Student’s *t*-test. The Mann–Whitney *U* test was performed for unpaired data that were not normally distributed. One-way and two-way analysis of variance with multiple-comparison testing were used when analyzing more than two groups. The log-rank test was used to compare survival between groups.

## RESULTS

### *C. rodentium* causes a lethal infection in *Il22*^−/−^ mice

**Fig 1:**
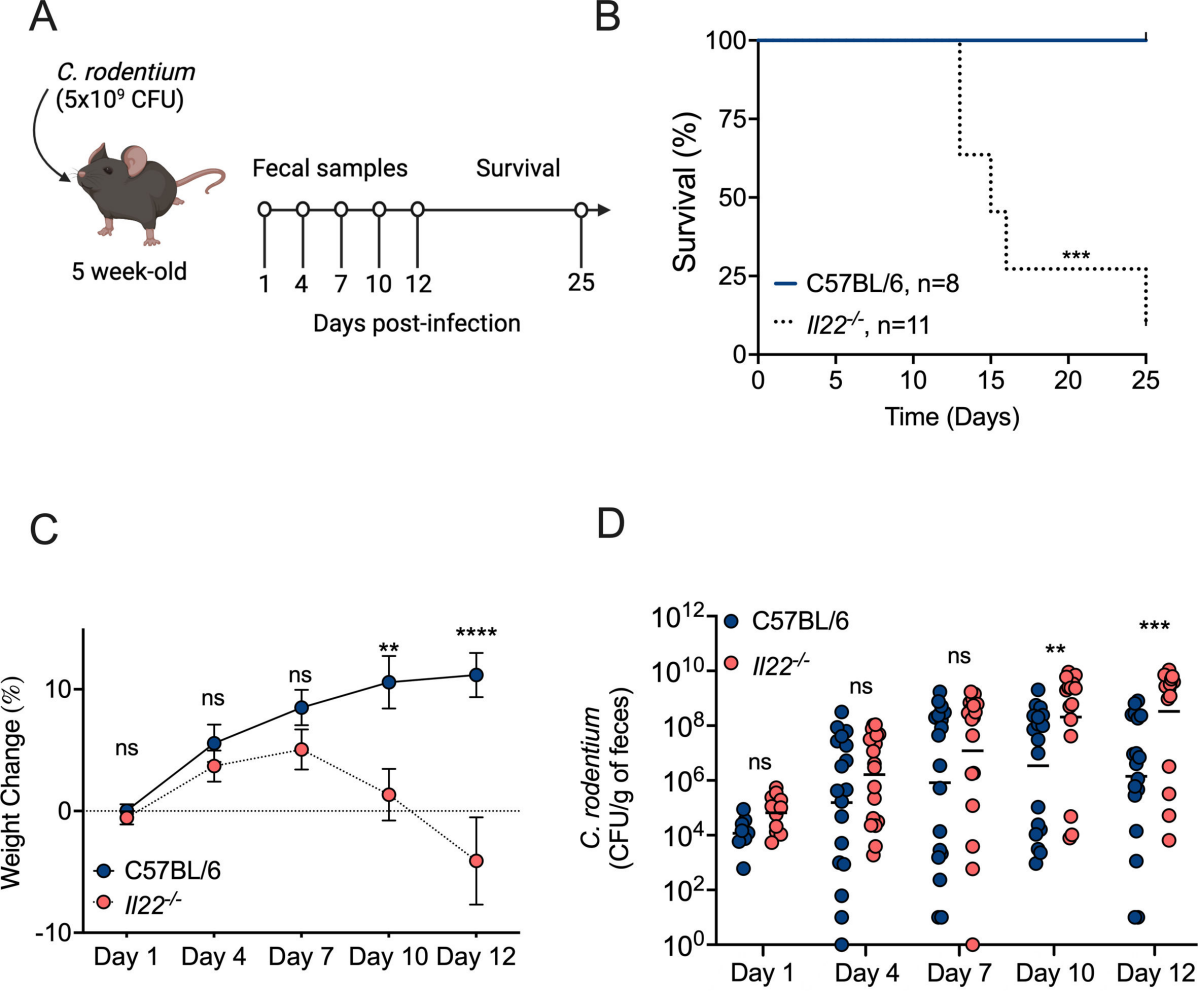
IL-22 confers protection during *C. rodentium* infection. (**A**) Scheme representing the *C. rodentium* infection timeline (created with Biorender). (**B**) Survival of WT and *Il22^−/^*^−^ mice infected with *C. rodentium*. (**C**) Weight course of mice infected with *C. rodentium*. (**D**) Fecal CFU of infected mice on Days 1, 4, 7, 10, and 12 of post-infection. Each circle represents an individual mouse. Bars represent the geometric mean. Significant differences are indicated by *P* < 0.01 (**) and *P* < 0.01 (***). ns = not significant.

*Il22*^−/−^ (cytokine knockout) and *Il22ra1*^−/−^ (receptor knockout) mice infected with *C. rodentium* exhibit heightened intestinal epithelial damage, increased systemic bacterial burden, and greater mortality (9, 16, 29). To confirm that *Il22*^−/−^ mice in our colony also displayed increased susceptibility to *C. rodentium* infection, we infected C57BL/6 *Il22^+/+^* and *Il22^−/^*^−^ cohoused littermate mice with *C. rodentium* and collected fecal samples to analyze colonization by the pathogen at Days 1, 4, 7, 10, and 12 of post-infection. Overall, we monitored the mice for 25 days of post-infection (Fig. 1A). All WT mice infected with *C. rodentium* were alive at Day 25, showed no weight loss, and exhibited a decline in the pathogen’s colonization level over time (Fig. 1B through D). In stark contrast, *Il22^−/^*^−^ mice infected with *C. rodentium* displayed remarkable susceptibility, with 91% lethality (using 20% wt loss or moribund behavior/appearance as a humane endpoint) by Day 25 (Fig. 1B). Moreover, these *Il22^−/^*^−^ mice showed significantly higher weight loss and colonization levels compared to WT controls (Fig. 1C and D). These findings align with prior studies and confirm that *C. rodentium* indeed induces a lethal infection in *Il22^−/^*^−^ mice bred in our animal facility.

**Fig 2:**
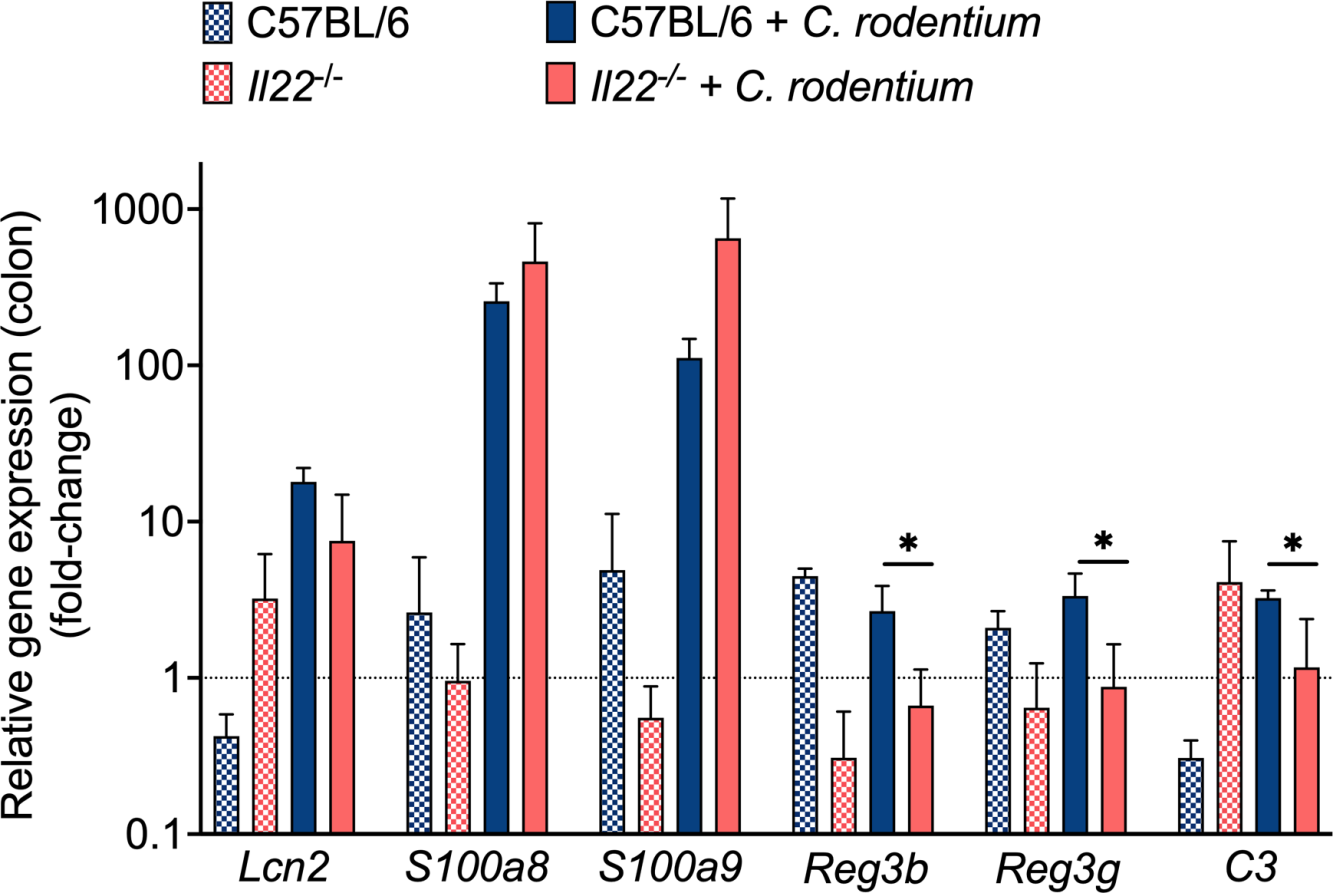
Expression of antimicrobial genes in WT and *Il-22^−/^*^−^ mice. RT-qPCR was used to determine the expression of *Lcn2*, *S100a8*, *S100a9*, *Reg3g*, *Reg3b,* and *C3* in the colon of uninfected WT and *Il22^−/^*^−^ mice, or 12 days after *C. rodentium* infection. WT infected, *n* = 4; *Il22^−/^*^−^ infected, *n* = 4; *Il22^−/^*^−^ mock, *n* = 2; WT mock, *n* = 2. Fold increase is expressed as the ratio between the infected WT or *Il22^−/^*^−^ mice and the respective mock-infected mice. Bars represent the geometric mean ± SD. Significant differences are indicated by *P* < 0.05 (*).

Because *Il22^−/^*^−^ mice started to succumb to *C. rodentium* infection beginning at Day 12 of post-infection (Fig. 1B), we selected this time point to analyze the expression of antimicrobial genes that are under IL-22 modulation (Fig. 2). The expression levels of *Lcn2*, which encodes for the antimicrobial protein lipocalin-2, and of *S100a8* and *S100a9*, whose gene products together form the antimicrobial protein calprotectin, were increased during infection but were comparable between WT mice and *Il22^−/^*^−^ mice. In contrast, expression of *C3* (encoding complement component C3) and of *Reg3b* and *Reg3g* (encoding the C-type antimicrobial lectins Reg3β and Reg3γ, respectively) was significantly lower in *Il22^−/^*^−^ mice than in WT mice (Fig. 2). Thus, when mice began to succumb to *C. rodentium* infection, there were differences in the expression levels of certain antimicrobial proteins that may contribute to host protection during *C. rodentium* infection.

### Mice deficient in calprotectin, lipocalin-2, or C3 do not succumb to *C. rodentium* infection

**Fig 3:**
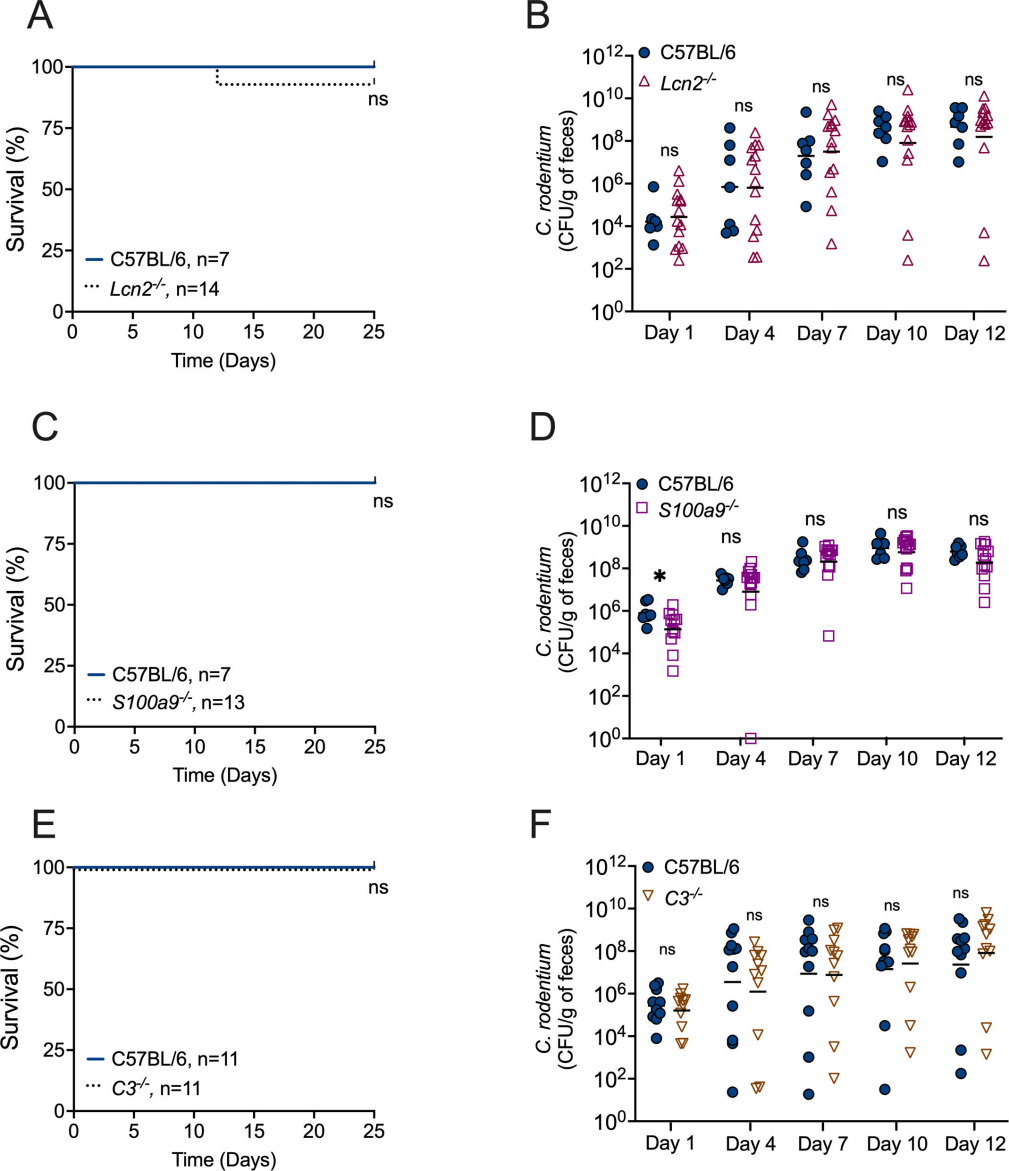
Mice lacking calprotectin, lipocalin-2, or C3 are resistant to *C. rodentium* infection. Survival course of WT and (**A**) *Lcn2^−/^*^−^ mice, (**C**) *S100a9^−/^*^−^ mice, and (**E**) *C3^−/^*^−^ mice infected with *C. rodentium*. Fecal CFU of (**B**) *Lcn2^−/^*^−^ mice, (**D**) *S100a9^−/^*^−^ mice, and (**F**) *C3^−/^*^−^ mice infected with *C. rodentium* at Days 1, 4, 7, 10, and 12 of post-infection. Each circle represents an individual mouse. Bars represent the geometric mean. ns = not significant.

Because *Il22^−/^*^−^ mice displayed lower induction of some antimicrobial genes in the gut, we investigated whether specific IL-22-dependent antimicrobial responses contributed to host protection during this infection. Lipocalin-2 is an antimicrobial protein known to inhibit bacterial growth by sequestering the siderophore enterobactin (20, 34), which some bacteria, including *C. rodentium*, utilize for acquiring iron from the environment. However, some bacterial pathogens, including *S. enterica* serovar Typhimurium, can acquire iron through alternative mechanisms and are therefore resistant to lipocalin-2 (18, 35). Since IL-22 induces lipocalin-2 expression by gut epithelial cells (18), we examined whether lipocalin-2 confers protection during *C. rodentium* infection. *Lcn2*^−/−^ mice were infected and monitored for *C. rodentium* colonization, weight loss, and survival over 25 days. In general, we did not observe significant differences in terms of survival (Fig. 3A; only one *Lcn2*^−/−^ mouse succumbed to infection), colonization levels (Fig. 3B), or weight loss (Fig. S1A) compared to WT mice. Thus, lipocalin-2 deficiency does not recapitulate the survival defect seen in *Il22*^−/−^ mice during *C. rodentium* infection.

Colonic epithelial cells stimulated with IL-22 also express the antimicrobial protein calprotectin (18), a heterodimer composed of S100A8 and S100A9, which is also highly expressed by neutrophils (36). Calprotectin exerts its antimicrobial activity by restricting metal nutrient availability to pathogens (36, 37). While some bacteria are susceptible to the antimicrobial activity of calprotectin (14, 38), others are resistant (39, 40). To investigate whether calprotectin contributes to protection against *C. rodentium* infection, we infected *S100a9*^−/−^ mice with *C. rodentium* and monitored mouse survival and *C. rodentium* colonization for 25 days. The infection outcome in *S100a9*^−/−^ mice closely resembled that of WT mice (Fig. 3C and D; Fig. S1B), indicating that *C. rodentium* is resistant to calprotectin and that *S100a9*^−/−^ mice do not phenocopy the survival impairment observed in *Il22*^−/−^ mice.

A previous study found that IL-22 plays a protective role during *Clostridium difficile* infection by modulating the expression of complement component C3, which, in turn, enhances phagocytosis (19). To investigate whether C3 also has a protective role during *C. rodentium* infection, we infected *C3*^−/−^ mice. All WT and *C3*^−/−^ mice survived for 25 days after *C. rodentium* infection (Fig. 3E), displayed comparable levels of *C. rodentium* colonization (Fig. 3F), and showed no signs of weight loss (Fig. S1C). Thus, the absence of C3 does not reduce mouse survival during *C. rodentium* infection.

### Mice deficient in the antimicrobial proteins REG3β and REG3γ survive infection with *C. rodentium*

**Fig 4:**
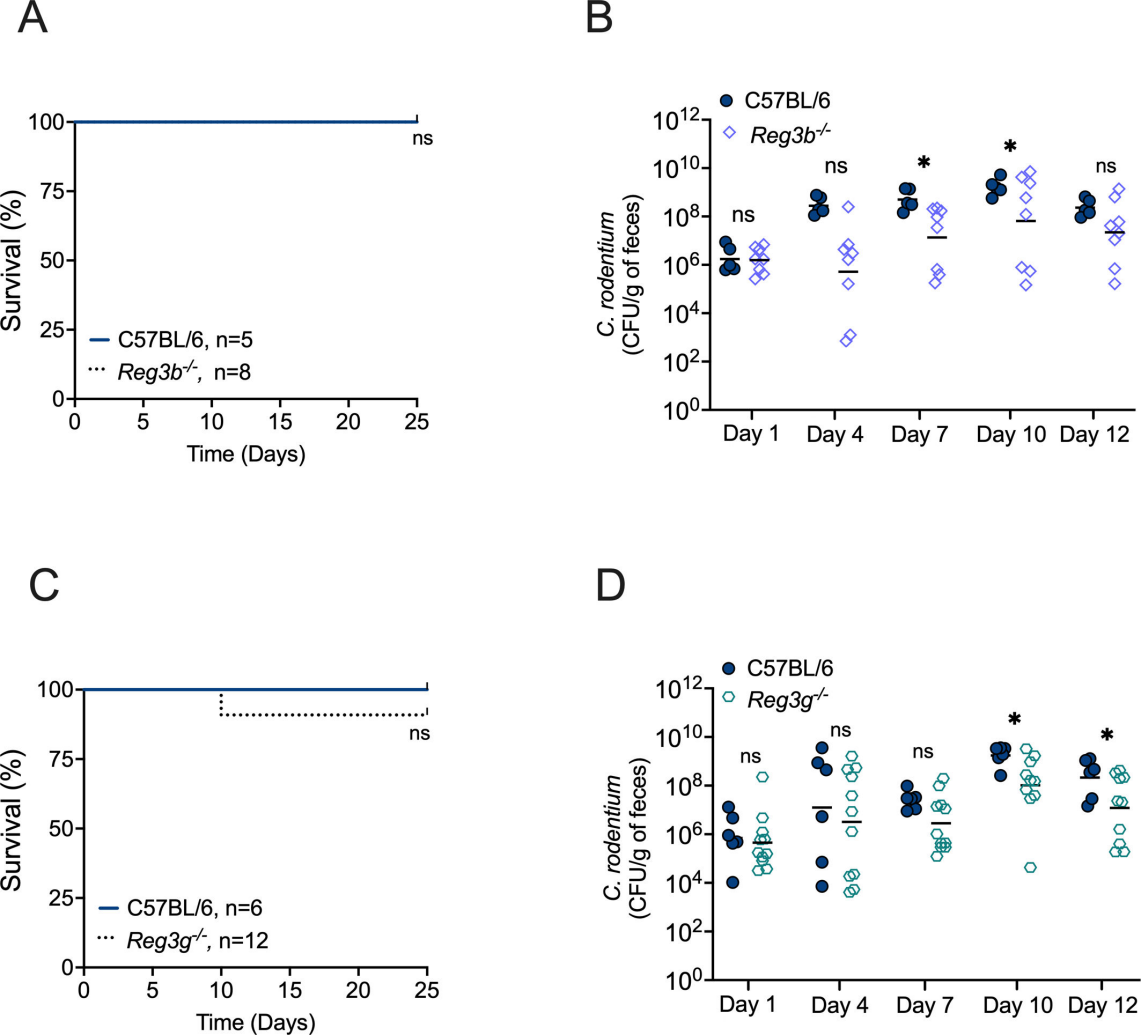
Mice lacking REG3β or REG3γ are resistant to *C. rodentium* infection. Survival course of WT and (**A**) *Reg3b^−/^*^−^ or (**C**) *Reg3g^−/^*^−^ mice infected with *C. rodentium*. Fecal CFU of (**B**) *Reg3b^−/^*^−^ and (**D**) *Reg3g^−/^*^−^ mice on Days 1, 4, 7, 10, and 12 of post-infection. Each circle represents an individual mouse. Bars represent the geometric mean. Significant differences are indicated by *P* < 0.05 (*). ns = not significant.

REG3β and REG3γ are two antimicrobial lectins secreted by Paneth cells and epithelial cells. They function by directly binding to bacterial membranes and are upregulated in response to IL-22 (9, 10, 13, 41–43). A previous study demonstrated that intraperitoneal injection of a recombinant mouse Reg3γ-Ig fusion protein (rmReg3γ) improved the survival of *Il22^−/^*^−^ mice (9). To assess the specific contribution of endogenous Reg3β and Reg3γ during *C. rodentium* infection, we infected *Reg3b*^−/−^ mice and *Reg3g*^−/−^ mice, monitoring colonization levels, weight loss, and survival. Mice lacking either Reg3β or Reg3γ did not exhibit an increased susceptibility to *C. rodentium* infection. With the exception of one *Reg3g*^−/−^ mouse, all survived until the 25-day experimental endpoint (Fig. 4A and C). Of note, *C. rodentium* colonization levels in both *Reg3b*^−/−^ and *Reg3g*^−/−^ mice were actually significantly lower than in WT mice on some days (Fig. 4B and D), suggesting that *C. rodentium* may exploit the expression of these antimicrobial proteins to compete with the microbiota. Nevertheless, no significant survival defect (Fig. 4A and C) nor weight loss (Fig. S1D and E) was observed relative to WT mice. Thus, Reg3β and Reg3γ, individually, are not essential for host survival during *C. rodentium* infection.

### *Il22*^−/−^ mice develop more severe colitis during *C. rodentium* infection

**Fig 5:**
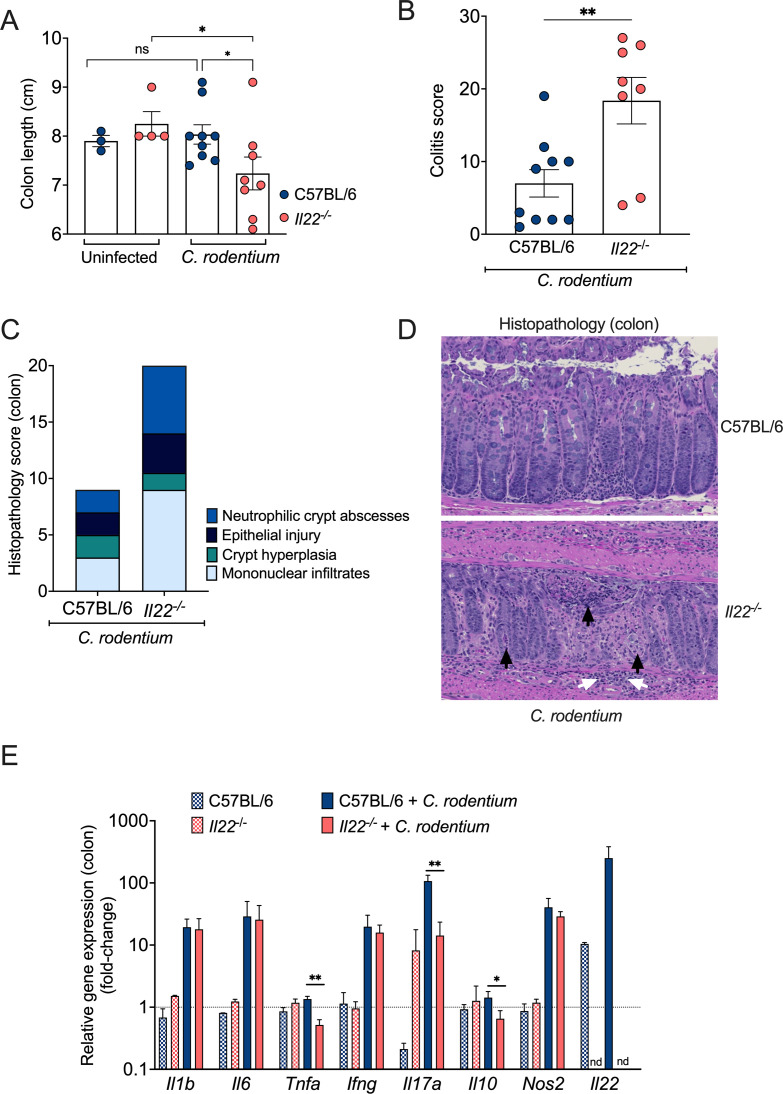
IL-22 is essential to ameliorate *C. rodentium*-induced inflammation in the recovery phase. (**A**) Colon length of infected and uninfected WT and *Il22^−/^*^−^ mice at Day 12 of post-infection. (**B**) Colitis scores of infected WT and *Il22^−/^*^−^ mice at Day 12 of post-infection. (**A, B**) Each circle represents an individual mouse. (**C**) Histopathology scores with individual subcategories at Day 12 of post-infection. Black arrows indicate crypt abscesses, and white arrows delineate submucosal inflammation. (**D**) Representative H&E-stained colon sections from mice infected with *C. rodentium*. (**E**) RT-qPCR was used to determine the expression of *Il1b*, *Il6*, *Tnfa, Ifng*, *Il17a, Il10, Nos2,* and *Il22* in the colon of uninfected WT and *Il22^−/^*^−^ mice, or 12 days after infection. WT infected, *n* = 4; *Il22^−/^*^−^ infected, *n* = 4; *Il22^−/^*^−^ mock, *n* = 2; WT mock, *n* = 2. Bars represent the geometric mean ± SD. Significant differences are indicated by *P* < 0.05 (*), *P* < 0.01 (**), and nd = not detected.

Our results indicate that the severity of disease observed in *Il22^−/^*^−^ mice infected with *C. rodentium* cannot be replicated in mice lacking particular antimicrobial responses modulated by IL-22. To explore alternative mechanisms of IL-22-mediated protection, we compared the inflammatory response in the colon of WT mice and *Il22^−/^*^−^ mice 12 days after *C. rodentium* infection, which is the time point at which mice began to succumb in our colony (Fig. 1B). Upon gross examination, infected WT mice exhibited colons of similar length to uninfected WT and *Il22^−/^*^−^ mice. In contrast, infected *Il22^−/^*^−^ mice had significantly shorter colons, indicative of inflammation (Fig. 5A). To further assess the degree of inflammation between the groups, we collected entire colons and prepared Swiss rolls for histopathological analysis. The total colitis score confirmed considerably higher inflammation in *Il22^−/^*^−^ mice compared to WT mice (Fig. 5B). This increased inflammation was characterized by enhanced epithelial injury and a substantial increase in mononuclear cell infiltrates and neutrophilic crypt abscesses (Fig. 5C and D). Furthermore, the higher colonic inflammation observed among infected *Il22^−/^*^−^ mice corresponded with reduced induction of *Il17a* (encoding IL-17A). Expression of *Il10* and *Tnfa* (encoding IL-10 and TNF-α, respectively) was modest but also lower in infected *Il22*^−/−^ mice. In contrast, expression levels of *lI1b*, *Il6*, *Ifng*, and *Nos2* (encoding IL-1β, IL-6, IFN-γ, and iNOS) were similarly increased in both WT and *Il22^−/^*^−^ mice at this time point (Fig. 5E). Together, these results indicate that IL-22 ameliorates colonic inflammation triggered by *C. rodentium* infection.

### *Il22*^−/−^ mice exhibit increased gut permeability and enhanced bacterial translocation during *C. rodentium* infection

**Fig 6:**
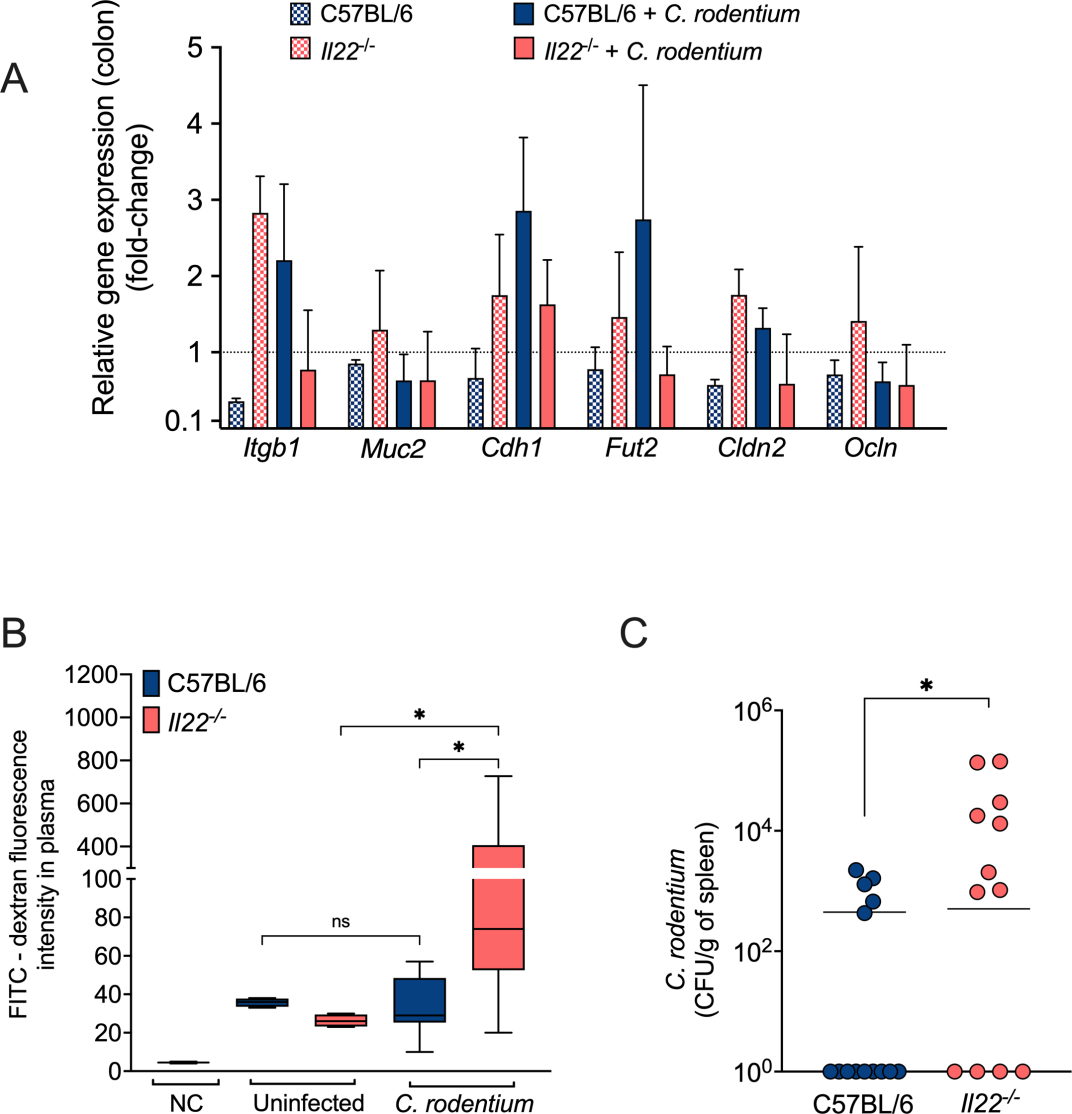
*Il-22^−/^*^−^ mice exhibit decreased expression of epithelial repair and integrity genes as well as altered barrier function during *C. rodentium* infection. (**A**) RT-qPCR was used to determine the expression of *Itgb1*, *Muc2*, *Cldn1*, *Fut2*, *Cldn2,* and *Ocln* in the colon of uninfected WT and *Il-22^−/^*^−^ mice, or 12 days after infection. WT infected, *n* = 4; *Il22^−/^*^−^ infected, *n* = 4; *Il22^−/^*^−^ mock, *n* = 2; WT mock, *n* = 2. Bars represent the geometric mean ± SD. (**B**) Intestinal permeability of *Il22^−/^*^−^ mice was determined by measuring FITC-dextran levels in the plasma of mice infected with *C. rodentium* at Day 12 of post-infection. Uninfected WT and *Il22^−/^*^−^ mice were used as baseline controls for intestinal permeability. Infected WT, *n* = 10; *Il-22^−/^*^−^ infected, *n* = 7; *Il22^−/^*^−^ mock, *n* = 2; WT mock, *n* = 4. Each box represents the median, and whiskers represent the minimum and maximum of FITC-dextran concentration in plasma. (**C**) *C. rodentium* CFU in the spleen 12 days of post-infection. Bars represent the geometric mean. Each circle represents an individual mouse. Significant differences are indicated by *P* < 0.05 (*).

Previous studies have shown that IL-22 plays a role in enhancing gut epithelial integrity and barrier function (9, 44). In light of this, we investigated whether the expression of genes known to be associated with gut integrity and permeability is altered in *C. rodentium*-infected *Il22^−/^*^−^ mice. Our data confirmed that expression of the integrin beta-1 gene *Itgb1*, which is linked to intestinal cell function and proliferation (45–47), was lower among infected *Il22^−/^*^−^ mice compared to WT mice (Fig. 6A). Additionally, the expression of some genes (*Cdh1*, cadherin-1, and *Cldn2*, claudin-2, but not *Ocln*, occludin) encoding tight junction and adherence junction proteins in the intestinal epithelium (28, 48–50) was also generally lower in infected *Il22^−/^*^−^ mice (Fig. 6A). Fucosyltransferase-2 and mucin-2 have important roles in intestinal epithelial function and have been associated with host resistance against *C. rodentium* (29, 51, 52). In our study, we observed no significant difference in *Muc2* expression between infected WT and *Il22^−/^*^−^ mice. Instead, *Fut2* expression was lower in infected *Il22^−/^*^−^ mice. Even though these changes in gene expression were small, overall they suggested that *Il22^−/^*^−^ mice have a more compromised intestinal epithelial barrier than WT mice during *C. rodentium* infection.

To assess whether gut permeability is functionally compromised in *Il22^−/^*^−^ mice, we conducted a FITC-dextran permeability assay. Twelve days after *C. rodentium* infection, WT and *Il22^−/^*^−^ littermate mice were administered 4-kD FITC-dextran by oral gavage. Four hours later, serum samples were collected to determine FITC fluorescence levels. In WT mice infected with *C. rodentium*, levels of FITC-dextran detected in the plasma were similar to those in uninfected mice (Fig. 6B), suggesting that the gut barrier was intact at this time point. In stark contrast, plasma FITC-dextran levels were significantly higher in *C. rodentium-*infected *Il-22^−/^*^−^ mice compared to both uninfected mice and infected WT mice (Fig. 6B). Additionally, at this time point, we recovered significantly higher *C. rodentium* CFU from the spleen of *Il22^−/^*^−^ mice compared to WT mice (Fig. 6C), providing further evidence of the increased permeability observed in *Il22^−/^*^−^ mice. Together, our data indicate that IL-22 plays a crucial role in maintaining intestinal barrier function during *C. rodentium* infection. The “leaky gut” phenomenon and increased pathogen dissemination likely represent key mechanisms through which IL-22 deficiency results in increased susceptibility to lethal *C. rodentium* infection of C57BL/6 mice.

## DISCUSSION

IL-22 is a cytokine that promotes host defense through various mechanisms, including the induction of antimicrobial responses and enhancement of the intestinal mucosal barrier (53). The cytokine is essential for clearance of *C. rodentium* and disease amelioration (9, 16, 29, 54) as *Il22^−/^*^−^ mice eventually succumb to the infection, despite a functional adaptive immune response (9).

Our findings align with previous studies demonstrating that IL-22 is essential for mice to survive *C. rodentium* infection (9, 16, 29, 54). In our study, *Il22^−/^*^−^ mice exhibited higher levels of *C. rodentium* colonization and increased colonic inflammation (Fig. 1A, 5B and C). At 12 days of post-infection, *Il22^−/^*^−^ mice began to succumb to the infection, whereas WT mice started to recover and cleared *C. rodentium* from the gut. Only one *Il22^−/^*^−^ mouse out of 11 infected survived to Day 25 of post-infection, while all infected WT mice survived (Fig. 1). *C. rodentium* infection of murine strain backgrounds that exhibit a mild disease phenotype, such as WT C57BL/6 mice, can be divided into four distinct phases (5). In the establishment phase (1–3 days of post-infection), the pathogen adapts to the gut environment. During the expansion phase (4–8 days of post-infection), *C. rodentium* proliferates and attaches to intestinal epithelial cells by expressing virulence factors. In the steady-state phase (8–12 days of post-infection), shedding reaches a plateau, with bacterial loads ranging between 10^8^ and 10^9^ CFU/g feces. Finally, in the clearance phase, typically occurring after 12 days of post-infection, the host begins to recover from the infection and clears the pathogen until no CFU can be detected (5). In the absence of IL-22, C57BL/6 mice fail to transition into the clearance phase. Instead of effectively clearing the infection, these mice succumb to the disease.

Although the pivotal role of IL-22 as a mediator of host protection and ultimately survival against *C. rodentium* infection has been established, our understanding of which host responses downstream of IL-22 contribute to this phenotype remains limited. During infection, many pathogens require the acquisition of metal nutrients to successfully colonize the host (35, 55–58). During *C. rodentium* infection, the metal-sequestering, IL-22-regulated antimicrobial proteins lipocalin-2 and calprotectin are highly expressed in the colon. Nevertheless, we found that mice lacking either of these proteins do not exhibit increased susceptibility to *C. rodentium* infection. In other words, a single deficiency of lipocalin-2 or calprotectin does not recapitulate the phenotype observed in *Il22^−/^*^−^ mice and further suggests that, *in vivo*, *C. rodentium* is resistant to metal sequestration by lipocalin-2 and calprotectin. Prior findings have demonstrated that *C. rodentium* is instead susceptible to heme limitation by the heme-binding protein hemopexin, whose expression is also regulated by IL-22. In the absence of hemopexin, *C. rodentium* can acquire heme iron more efficiently and achieve greater dissemination (16).

The complement system is an important host response that eliminates pathogens by promoting their recognition, ingestion, and destruction by phagocytic cells (59). IL-22 has been shown to control infections by inducing the complement system, resulting in enhanced bacterial phagocytosis through the modulation of complement C3 expression (19). In our study, we found that young adult (5–9 weeks of age) *C3*^−/−^ mice exhibited resistance to *C. rodentium* infection similar to that of WT mice. A separate study reported that young (21-day-old) *C3*^−/−^ mice were found to be more susceptible to *C. rodentium* infection than age-matched WT mice (60). Thus, although C3 deficiency does not lead to lethal *C. rodentium* infection in adult mice, the role of C3 may be more critical in early life.

IL-22 regulates the expression of the C-type lectins Reg3β and Reg3γ (9, 10). Members of the Reg3 protein family exert direct antibacterial activity by binding to components of bacterial membranes, including peptidoglycan (10, 61) and lipid A (41), which assists the host in controlling infections (62, 63). During *C. rodentium* infection, the endogenous intraperitoneal administration of Reg3γ was sufficient to partially alleviate mortality of *Il22^−/^*^−^ mice (9). However, another study showed that *Reg3g*^−/−^ mice exhibited resistance similar to that of WT mice during *C. rodentium* infection (29). Our findings are in alignment with the latter study, as neither *Reg3b*^−/−^ nor *Reg3g*^−/−^ mice in our colony succumbed to *C. rodentium* disease. These collective observations suggest that the antimicrobial activity of Reg3 family proteins in the gut may not be the primary mechanism by which IL-22 mediates host defense against *C. rodentium* infection.

In addition to inducing antimicrobial responses, IL-22 also modulates intestinal barrier integrity, thereby ameliorating colonic inflammation (44, 54, 64). Consistent with these reports, we found that *Il22^−/^*^−^ mice exhibited more severe inflammation than WT mice following *C. rodentium* infection. Although several proinflammatory cytokine genes, including *Il1b*, *Il6*, and *Ifng*, were similarly induced in both *Il22^−/^*^−^ and WT mice, reduced expression of other cytokines, particularly pro-inflammatory *Il17a*, may help to explain the increased pathology observed in *Il22^−/^*^−^ mice. Moreover, the higher colonic inflammation in *Il22^−/^*^−^ mice could potentially be attributed to tissue damage and inadequate tissue repair. Key genes responsible for maintaining epithelial integrity and promoting recovery, such as those encoding claudin-2, e-cadherin, and β1-integrin, displayed reduced expression during infection in *Il22^−/^*^−^ mice. Functionally, disruption of the epithelial barrier in *Il22^−/^*^−^ mice was confirmed by a gut permeability assay, wherein elevated FITC-dextran levels were observed in the serum, and by increased *C. rodentium* translocation to the spleen.

Using a combination of three probes with different diameters, a prior study demonstrated that the pore and leak pathway permeabilities increased early (Day 2) during *C. rodentium* infection, whereas the unrestricted pathway, due to tissue damage, was responsible for the increased permeability at later time points (Day 6 and after) (28). Thus, the increased permeability that we detected with 4-kD FITC-dextran at Day 12 of post-infection in *Il22*^−/−^ mice may be due to the leak and/or unrestricted pathways, although the apparent tissue damage (Fig. 5) suggests the unrestricted pathway. Nevertheless, future studies are necessary to precisely elucidate the mechanism underlying the higher permeability in *Il22*^−/−^ mice. Overall, our findings are consistent with previous studies that emphasize the role of IL-22 in promoting and maintaining gut epithelial integrity (29, 44, 54, 64). Notably, mice lacking claudin-2 were found to be highly susceptible to *C. rodentium* infection (28), albeit not to the level of *Il22^−/^*^−^ mice.

Another gene with reduced expression in infected *Il22^−/^*^−^ mice is *fut2*, which encodes a 1,2-fucosyltransferase involved in epithelial glycosylation (65)The IL-22 receptor IL-22RA1 has been shown to promote intestinal fucosylation, thereby enhancing colonization resistance by increasing anaerobic commensal diversity. This, in turn, prevents the translocation of opportunistic pathogens during inflammation and infection, ultimately supporting host recovery (29). The study of Pickard et al. (66) further supports these observations, demonstrating that fucose is liberated and metabolized by the gut microbiota in response to microbial stimuli. This process fosters the integration of host and gut microbes, providing protection by decreasing the expression of bacterial virulence genes and enhancing host tolerance to *C. rodentium*. Taken together, these studies underscore the important role of IL-22 and Fut2 in maintaining proper fucosylation levels to enhance host defense against pathogens such as *C. rodentium*. Thus, the observed susceptibility to *C. rodentium* infection in *Il22^−/^*^−^ mice may also be attributed, at least in part, to the diminished expression of Fut2.

Our findings collectively suggest that the heightened susceptibility of *Il22^−/^*^−^ mice to *C. rodentium* infection is attributable to a combination of host defense mechanisms. It is worth noting that our study was designed to identify whether particular IL-22-mediated antimicrobial responses were responsible for host survival during *C. rodentium* infection; as such, mice lacking one of these antimicrobial responses might exhibit differences in bacterial colonization, host gene expression, or inflammation that were not assayed for here. Moreover, for future studies, it would be important to evaluate the role of the gut microbiota and its possible alterations in these knockout mice. In an attempt to control for this, we used cohoused WT littermates for all experiments with *Il22*^−/−^ mice, which is the best approach to standardize the microbiota. The other knockout mice were instead cohoused with WT mice purchased from the Jackson Laboratory, as our goal was to eventually compare littermates from the colonies from which we had a survival phenotype. In the future, the use of cohoused littermate WT mice for all colonies would be important for teasing out possible differences in the host response to *C. rodentium*.

Taken together, our study expands on and reinforces the essential role of IL-22 for the resolution of *C. rodentium* infection, which is not solely dependent on a single antimicrobial response (calprotectin, lipocalin-2, Reg3β, Reg3γ, or C3) induced by IL-22. Additionally, our research broadens our understanding of the role IL-22 plays in maintaining and repairing the integrity of the gut epithelial barrier in the recovery phase of infection. This process emerges as necessary for the host’s ability to survive *C. rodentium* infection.

## SUPPLEMENTAL MATERIAL

Supplemental material iai.00099-24-s0001.docx Figure S1 and Table S1.

ASM does not own the copyrights to Supplemental Material that may be linked to, or accessed through, an article. The authors have granted ASM a non-exclusive, world-wide license to publish the Supplemental Material files. Please contact the corresponding author directly for reuse.
