# Supplementary material for: IL-22-dependent responses and their role during Citrobacter rodentium infection
Source: Infect Immun. 2024 Apr 1;92(5):e00099-24. doi: 10.1128/iai.00099-24 (PMC11075456; doi:10.1128/iai.00099-24)
Supplement: Supplemental material — Figure S1 and Table S1. [file iai.00099-24-s0001.docx]

**Supplemental Figure 1**

**
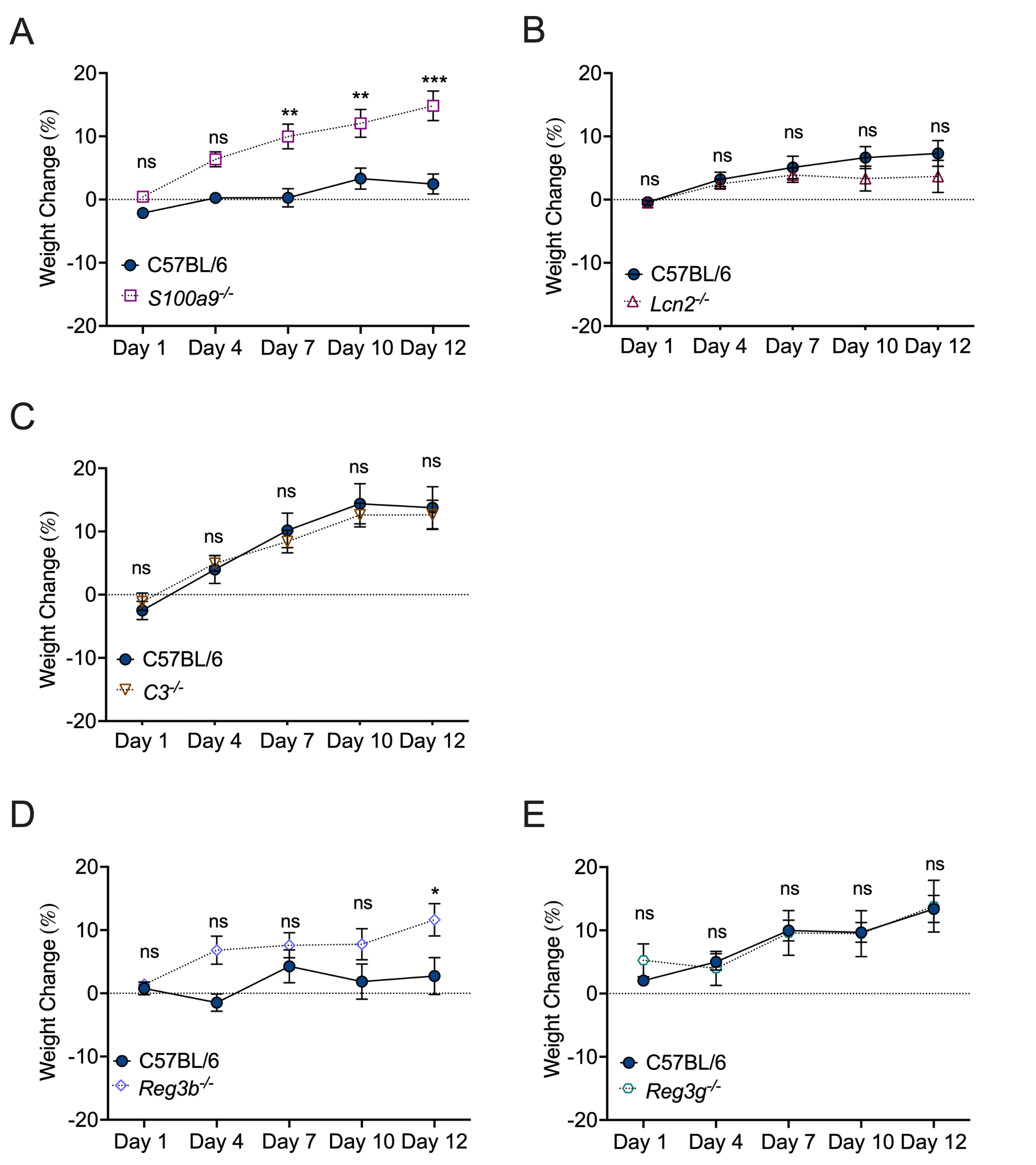
**

**Supplemental Figure 1.** Weight course of WT and **(A)** *S100a9^-/-^*, **(B)** *Lcn2^-/-^*, **(C)** *C3^-/-^* , **(D)** *Reg3b^-/-^*, and **(E)** *Reg3γ^-/-^* mice infected with *C. rodentium*. Bars represent the geometric mean ± SD. Significant differences are indicated by p<0.05 (*), p<0.01 (**), and p<0.01 (***). ns = not significant.

**Supplemental Table 1 - Primers used in this study**

| **Target** | **Forward primer (5’ – 3’)** | **Reverse primer (5’ – 3’)** | **Reference** |
| --- | --- | --- | --- |
| *Cldn1* | TCCTTGTTCGGCTATGTGTC | GGCATGCACCTAAGAATCAG | [(28)](https://www.zotero.org/google-docs/?F1W3gW) |
| *Cldn2* | GGCTGTTAGGCACATCCAT | TGGCACCAACATAGGAACTC | [(28)](https://www.zotero.org/google-docs/?M0wauy) |
| *Fut2* | TGCACTGGCCAGGATGAA | GCGCTAGAGCGTTGTGCAT | [(29)](https://www.zotero.org/google-docs/?Y1T6Q4) |
| *Ifng* | TCAAGTGGCATAGATGTGGAAGAA | TGGCTCTGCAGGATTTTCATG | [(67)](https://www.zotero.org/google-docs/?Vdk9Id) |
| *Il1b* | ATGAGAGCATCCAGCTTCAA | TGAAGGAAAAGAAGGTGCTC | [(68)](https://www.zotero.org/google-docs/?O8MX8A) |
| *Il6* | GAGGATACCACTCCCAACAGACC | AAGTGCATCATCGTTGTTCATACA | [(67)](https://www.zotero.org/google-docs/?X19g2c) |
| *Il10* | GGTTGCCAAGCCTTATCGGA | ACCTGCTCCACTGCCTTGCT | [(67)](https://www.zotero.org/google-docs/?SF9vTV) |
| *Il22* | GGCCAGCCTTGCAGATAACA | GCTGATGTGACAGGAGCTGA | [(9)](https://www.zotero.org/google-docs/?O1Nfkc) |
| *Itgb1* | AGTGCTCCCACTTCAATCTCACCA | TCTCCTTGCAATGGGTCACAGGAT | [(28)](https://www.zotero.org/google-docs/?r0ozaj) |
| *Muc2* | GCTGACGAGTGGTTGGTGAATG | GATGAGGTGGCAGACAGGAGAC | [(30)](https://www.zotero.org/google-docs/?eLzRIo) |
| *Nos2* | TTGGGTCTTGTTCACTCCACGG | CCTCTTTCAGGTCACTTTGGTAGG | [(69)](https://www.zotero.org/google-docs/?cihZB2) |
| *Ocln* | GCTGTGATGTGTGTGAGCTG | GACGGTCTACCTGGAGGAAC | [(28)](https://www.zotero.org/google-docs/?LZLqQJ) |
| *Reg3b* | ATGGCTCCTACTGCTATGCC | GTGTCCTCCAGGCCTCTTT | [(9)](https://www.zotero.org/google-docs/?MJccNk) |
| *Reg3g* | ATGGCTCCTATTGCTATGCC | GATGTCCTGAGGGCCTCTT | [(9)](https://www.zotero.org/google-docs/?3HmkUF) |
| *S100A8* | CCATCGCAAGGAACTCCTCGAAG | ACAAGGAAATCACCATGCCCTCTAC | Raffatellu Lab |
| *S100A9* | ATTCAGACAAATGGTGGAAGCACAG | CATCAGCATCATACACTCCTCAAAGC | Raffatellu Lab |
| *Tnfa* | CATCTTCTCAAAATTCGAGTGACAA | TGGGAGTAGACAAGGTACAACCC | [(67)](https://www.zotero.org/google-docs/?Ma4DDy) |

**Supplemental References**

67. Overbergh L, Giulietti A, Valckx D, Decallonne R, Bouillon R, Mathieu C. 2003. The use of real-time reverse transcriptase PCR for the quantification of cytokine gene expression. J Biomol Tech 14:33–43.

68. Wan Z, Fan Y, Liu X, Xue J, Han Z, Zhu C, Wang X. 2019. NLRP3 inflammasome promotes diabetes-induced endothelial inflammation and atherosclerosis. Diabetes Metab Syndr Obes 12:1931–1942. <https://doi.org/10.2147/DMSO.S222053>

69. Godinez I, Haneda T, Raffatellu M, George MD, Paixão TA, Rolán HG, Santos RL, Dandekar S, Tsolis RM, Bäumler AJ. 2008. T cells help to amplify inflammatory responses induced by Salmonella *enterica* serotype Typhimurium in the intestinal mucosa. Infect Immun 76:2008–2017. <https://doi.org/10.1128/IAI.01691-07>
